# A retroviral mutagenesis screen identifies *Cd74 *as a common insertion site in murine B-lymphomas and reveals the existence of a novel IFNγ-inducible Cd74 isoform

**DOI:** 10.1186/1476-4598-9-86

**Published:** 2010-04-23

**Authors:** Magdalena Pyrz, Bruce Wang, Matthias Wabl, Finn Skou Pedersen

**Affiliations:** 1 Department of Molecular Biology, Aarhus University, Aarhus, DK-8000, Denmark; 2 Picobella, LLC, 863 Mitten Road, Suite 101, Burlingame, CA-94010, USA; 3 Department of Microbiology and Immunology, University of California, San Francisco, CA-94143, USA

## Abstract

**Background:**

Insertional mutagenesis screens in the mouse are an acknowledged approach to identify genes involved in the pathogenesis of cancer. The potential of these screens to identify genes causally involved in tumorigenesis is not only limited to the murine host, but many of these genes have also been proven to be involved in the oncogenic process in man.

**Results:**

Through an insertional mutagenesis screen applying murine leukemia viruses in mouse, we found that *Cd74 *was targeted by proviral insertion in tumors of B-cell origin. This locus encodes a protein playing crucial roles in antigen presentation and B-cell homeostasis, and its deregulation is often associated with cancer in man. The distribution of insertions within the *Cd74 *locus prompted the identification of an alternative transcript initiated in intron 1 of *Cd74 *encoding an N-terminally truncated Cd74 isoform in tissues from un-infected mice, and transcriptional activation assays revealed a positive effect on the novel intronic promoter by a formerly described intronic enhancer in the *Cd74 *locus. Furthermore, we show that the new Cd74 isoform is IFNγ inducible and that its expression is differentially regulated from the canonical Cd74 isoform at the transcriptional level.

**Conclusions:**

We here identify *Cd74 *as a common insertion site in murine B-lymphomas and describe a novel IFNγ-inducible murine Cd74 isoform differentially regulated from the canonical isoform and expressed under the control of an intronic promoter. The distribution and orientation of proviral insertion sites within the *Cd74 *locus underscores the causal involvement of the isoforms in the murine B-lymphomagenic process.

## Background

Cd74 (CD74 in man) is a non-polymorphic type II membrane-spanning glycoprotein, which was originally identified as being associated with antigen presentation by dimeric major histocompatibility complex class II (MHCII) molecules. The roles played by Cd74 in antigen presentation span from chaperoning MHCII dimers in their proper folding, preventing premature antigenic peptide loading in the ER, and promoting ER egress of MHCII dimers, to targeting these complexes to endocytic compartments [1-3]. However, Cd74 is also required for follicular B-cell maturation as well as maintenance of the follicular and marginal zone B-cell pools [4,5], and this action is independent of the MHCII-chaperonic activity of Cd74 [6]. The vast majority of MHCII-Cd74 complexes are diverted to the endocytic system, but surface expression of a small proportion of cellular Cd74 can indeed be detected on B-cells independently of concomitant class II expression [7-9]. These Cd74 cell surface molecules reveal high affinity binding to the pro-inflammatory cytokine macrophage migration inhibitory factor (MIF), and together with the signaling component of the MIF-Cd74 receptor complex (CD44) transmit MIF-mediated signaling [10,11]. Activation of cell surface Cd74 by MIF induces a signaling cascade leading to Cd74 intra-membrane cleavage, release of the N-terminal cytoplasmic part of Cd74, NF-κB activation, and ultimately, increased expression of the anti-apoptotic factor Bcl-2 [12-15]. This signaling cascade defines Cd74 as a survival receptor enhancing the survival of mature B-cells. Furthermore, Cd74 was also shown to act as a regulator of dendritic and B-cell motility [16].

In normal tissues, Cd74 is expressed on B cells, monocytes, macrophages, dendritic cells and epithelial cells of endodermal and mesodermal origin [17]. The transcriptional control elements for the murine *Cd74 *locus are composed of a promoter with common regulatory elements such as TATA-box, Sp1 site, CCAAT-box, and an NF-κB responsive element as well as an upstream enhancer with elements corresponding to promoter elements in MHCII [18]. Additionally, two distinct intronic enhancers are found in intron 1 [19,20]. In mouse differential splicing gives rise to two different isoforms, p31 and p41, with the p41 isoform harboring an additional exon (exon6b) as compared to p31 [21] whereas in man differential splicing combined with alternative translational start sites gives rise to four distinct CD74 isoforms [22,23]. The mRNA transcripts for human p41 and p43, the longer isoform translated from an upstream ATG, comprise 10% of the total CD74 transcript pool [22]. Cd74 is involved in many different scenarios, however analysis of Cd74 function in transgenic mice expressing exclusively one of the two isoforms indicates that in most respects the two isoforms can be regarded as functionally redundant [24-26].

Given its diverse functions in B-cell homeostasis it is not surprising that human CD74 is strongly expressed in a variety of B-cell lymphomas as well as many cell lines derived thereof [27-29]. B-cell chronic lymphocytic leukemia (B-CLL) is characterized by a progressive accumulation of B-lymphocytes in peripheral blood, lymphoid organs, and bone marrow, due to decreased apoptosis of this cell population. Evaluation of CD74 function in B-cells purified from the peripheral blood of B-CLL patients revealed that cell surface stimulation of CD74 initiated a signaling cascade leading to promotion of cell survival [30]. Many studies have furthermore demonstrated CD74 expression in various non-hematological cancers including gastric, colon, lung, and renal epithelial cancers [31-34], and moreover has the elevated expression level in several cancers served as a marker for tumor progression and/or poor clinical outcome [35]. The selective expression pattern of CD74 in neoplastic processes combined with the dynamics of internalization of cell surface CD74 molecules have brought CD74 forward as an attractive target for monoclonal antibody-based therapy [35]. In that connection, preclinical studies revealed that a humanized form of a murine Cd74 antibody is able to effectively inhibit tumor growth and yielded marked survival improvements in severe combined immunodeficiency mouse xenograft models of non-Hodgkin lymphomas and multiple myeloma, respectively [36]. Currently, phase I and phase I/II trials are underway in patients with B-cell non-Hodgkin lymphomas and B-CLL, and one including patients with multiple myeloma has been completed [37].

Proviral tagging is a widely applied and efficient tool in the discovery of oncogenes relying on the mechanism of retroviral insertional mutagenesis [38,39]. In an attempt to broaden the understanding of the causative genetic alterations in hematopoietic tumors, we have used the inbred NMRI mouse strain infected with the non-acutely transforming ecotropic murine leukemia viruses (MLVs) Akv and SL3-3, giving rise to models of B- and T-cell tumorigenesis, respectively [40,41]. We here report of the identification of *Cd74 *as a novel common insertion site in retrovirally induced murine B-lymphomas, thereby providing a strong genetic indication for causal involvement of this locus in B-lymphomagenesis. Prompted by the distribution and orientation of insertion sites within the *Cd74 *locus a hitherto uncharacterized intronic *Cd74 *promoter was identified in tissues from un-infected mice leading to expression of an N-terminally truncated Cd74 isoform. The distinct intronic promoter is positively regulated by the 3' *Cd74 *intronic enhancer and the expression of the novel Cd74 isoform is IFNγ responsive in analogy with the canonical Cd74 isoform.

## Results

### *Cd74 *is a frequently targeted novel common insertion site

**Figure 1:**
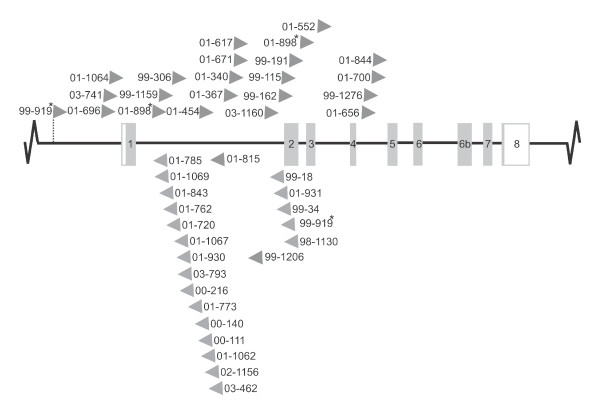
**Integration pattern in the *Cd74 *locus**. Shaded boxes indicate coding sequences and open boxes non-coding regions, numbers represent tumor IDs, and arrowheads indicate proviral orientation in relation to transcriptional direction. Asterisks mark two tumors (99-919 and 01-898) each harboring two integrations.

In a retroviral screen of app. 2400 tumors, 44 proviral insertion sites were identified within the locus encoding Cd74, with tumors harboring only a single integration in *Cd74 *per tumor in all but two cases. All insertions were found exclusively upstream of exon 5, displaying no general orientation preference, within a small window of 8.7 kb (Figure 1).

The screen resulted in about 7800 retroviral tags and assuming random distribution of these in the 3.2 gb murine genome, this would result in app. one integration per 410 kb in this screen, leading to an expected frequency of ~ 0.02 of randomly tagging the *Cd74 *locus which spans app. 9 kb. Therefore, identification of 44 integration sites within this small region is ~ 2200 times higher than expected if the locus were hit merely by chance. Furthermore, whereas the insertions in *Cd74 *co-transcriptional orientation were dispersed over the entire region upstream exon 5, all the proviral insertions in opposite transcriptional orientation to *Cd74 *clustered within intron 1 and upstream intron 2 (insertions in tumor 01-931, 99-34, the downstream insertion in 99-919, and 98-1130 were in exon 2, see Figure 1). This clearly indicates a selective preference for integrated proviruses of inverse transcriptional directionality to be found within intron 1. Collectively, these observations of high frequency and non-randomness in insertional position and orientation serve as powerful genetic arguments of positive selection for *Cd74 *insertions during lymphomagenesis.

The Retroviral Tagged Cancer Gene Database (RTCGD) collects data from multiple high-through-put retroviral insertional screens, enabling cross-screen searches for common insertion sites (CIS) [42,43]. Although the database currently contains nearly 7000 retroviral insertion sites *Cd74 *could not be found in RTCGD as tagged by insertional integration.

### Targeting of the *Cd74 *locus is specific for B-lymphomagenic MLVs

The tumors included in this screen were induced by Akv, SL3-3 and mutants hereof and the tumor bank consisted of app. equal sample sizes of tumors induced by MLVs possessing a B-lymphomagenic potential and T-lymphomagenic MLVs, respectively. As can be seen in table 1, 40 of the 42 tumors were induced by viruses of the Akv type possessing a strictly B-lymphomagenic potential. The remaining two tumors were induced by viruses with the B-lymphomagenic potential being one among others (SL3-3(AML1dm) [44] and Akv/SL3-3 TM (Unpublished)). Thus, despite the composition of the tumor bank, tumors with integration in *Cd74 *shared the common characteristic of being induced by B-lymphomagenic viruses.

**Table 1:** Tumor panel with integrations in the *Cd74 *locus

Tumor ID and tissue^a^	Virus variant^b^	Lymphomagenic potential of virus variant	Lymphoma incidence n/total	Mean latency in days^c^	Latency in days^d^
01-340s	Akv wt	B^e^	36/39	185 ± 26	194
01-367s	Akv wt	B^e^	36/39	185 ± 26	190
01-617s	Akv wt	B^e^	36/39	185 ± 26	251
99-919m	Akv1-99 wt	B^e^	67/70	181 ± 26	150
99-1206c	Akv1-99 wt	B^e^	67/70	181 ± 26	209
01-454s	Akv1-99 wt	B^e^	67/70	181 ± 26	198
99-34m	Akv PBS-Arg	B^f^	19/19	178 ± 17	172
99-162c	Akv PBS-Gln	B^f^	23/23	182 ± 27	204
99-18m	Akv PBS-Lys	B^g^	19/20	206 ± 30	172
99-115c	Akv PBS-Lys	B^g^	19/20	206 ± 30	204
99-191t	Akv PBS-Lys	B^g^	19/20	206 ± 30	225
98-1130s	Akv PBS-Pro	B^f^	20/20	161 ± 36	131
01-1064s	Akv1-99 (mAML1+mEgre)	B^h^	45/46	199 ± 35	232
01-671s	Akv1-99 (mEts)	B^h^	43/44	182 ± 27	163
01-700s	Akv1-99 (mEts)	B^h^	43/44	182 ± 27	169
01-762s	Akv1-99 (mEts)	B^h^	43/44	182 ± 27	177
01-931s	Akv1-99 (mEts)	B^h^	43/44	182 ± 27	204
99-1159s	Akv1-99NF1m1	B^i^	16/17	187 ± 19	199
01-656s	Akv1-99NF1m2	B^i^	44/44	182 ± 28	156
01-696s	Akv1-99NF1m2	B^i^	44/44	182 ± 28	162
01-785c	Akv1-99NF1m2	B^i^	44/44	182 ± 28	177
01-815c	Akv1-99NF1m2	B^i^	44/44	182 ± 28	181
01-843s	Akv1-99NF1m2	B^i^	44/44	182 ± 28	184
01-1067s	Akv1-99NF1m2	B^i^	44/44	182 ± 28	218
01-1069s	Akv1-99mEa/s	B^h^	47/47	165 ± 23	183
01-844s	Akv1-99mEa/s	B^h^	47/47	165 ± 23	149
01-898s	Akv1-99mEa/s	B^h^	47/47	165 ± 23	158
99-306t	SL3-3(AML1dm)	Mixed, incl. B ^j^	36/42	NA	175
01-552s	Akv1-99 (mAML1)	B^h^	43/45	126 ± 40	162
01-720s	Akv1-99 (mEgre)	B^h^	50/50	148 ± 47	185
03-462s	Akv1-99 (mEgre)	B^h^	41/42	160 ± 27	154
01-773s	Akv1-99 (mAML1+mGR)	B^h^	53/53	190 ± 31	203
01-1062m	Akv1-99 (mAML1+mGR)	B^h^	53/53	190 ± 31	245
03-741s	Akv1-99 (mEgre+mEa/s)	B^h^	41/41	183 ± 38	199
01-930s	Akv1-99 (mEts)	B^h^	43/44	182 ± 27	204
03-793s	Akv1-99 (mGr)	B^h^	40/41	145 ± 28	164
00-140n	Akv SA' gag mutant (EH)	B^k^	17/18	184 ± 34	205
00-111n	Akv SA' gag mutant (CD)	B^k^	17/19	201 ± 30	188
02-1156m	Akv/SL3-3TM	Ongoing work	23/23	139 ± 23	115
03-1160s	Akv1-99 (mGR+mEa/s)	B^h^	46/49	186 ± 26	240
00-216m	Akv SA' gag mutant (EH)	B^k^	17/18	184 ± 34	232
99-1276s	Akv SA' gag mutant (CDH)	B^k^	14/16	190 ± 46	163

^a^S indicates spleen; m, mesenteric lymph node; c, cervical lymph nodes; t, thymus; and n, lymph node of unspecified position. ^b^Indicate the MLV injected into inbred NMRI mice for tumor induction. ^c^Indicates the mean latency for tumor induction in mice infected with the same MLV as the one giving rise to the listed tumor with integration in *Cd74*. ^d^Indicates the age of the mouse harboring the listed tumor at the point of cervical dislocation and tumor isolation. The pathogenic potential of these viruses were determined in the following studies; ^e^[41], ^f^(Unpublished), ^g^[48], ^h^[46], ^i^[68], ^j^[44], and ^k^[67].

**Figure 2:**
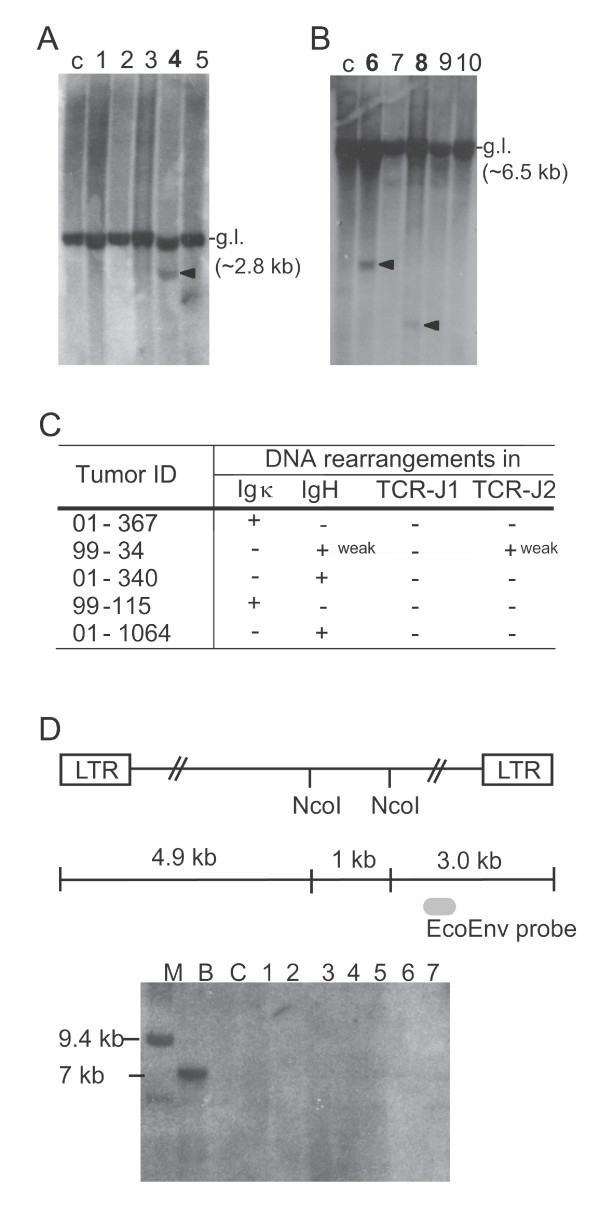
**Southern blotting analysis**. **(A) **and **(B) **Tumor DNA was analyzed by Southern blot hybridization using probes detecting germline (g.l.) and rearranged configurations (arrowhead) of the Igκ locus (A), IgH locus (B) and the TCRβ chain (data not shown). DNA from spleen from un-injected NMRI mice was included as control (c) and tumors with rearrangements are indicated in bold (lane 4 in (A): tumor ID 99-115, lane 6 in (B): 01-340, and lane 8 in (B): 99-34). **(C) **List of all tumors with detected rearrangements in any of the analyzed loci. **(D) **Evaluation of integration site clonality by Southern blotting analysis. Tumor DNA was analyzed by Southern blot hybridization using an ecotropic envelope-specific probe, the position of which is indicated. The blot exemplifies an analysis of DNA from seven tumors harboring integration in *Cd74 *(lanes marked by numbers), DNA from spleen of un-infected NMRI mice (lane denoted C), and DNA from spleen of un-infected BALB/c mice (lane denoted B). The lane denoted M contains the marker. BALB/c DNA contains one copy of an endogenous ecotropic MLV (which upon *Nco*I digestion and hybridization with this probe yields a band of app. 7 kb), thus serving as a positive control for the envelope probe, whereas NMRI was included as a negative control as it does not harbor endogenous ecotropic MLVs. LTR denotes the long terminal repeats of integrated proviruses.

Rearrangements within immunoglobulin (Ig) and T-cell receptor (TCR) genes, normally occurring during B- and T-cell development, are useful diagnostic markers for determining the cell lineage of lymphomas. In accordance with a common B-lymphomagenic potential of the viruses applied for tumor induction of *Cd74*-targeted tumors and the tumor target tissue, no general pattern of rearrangements in the TCRβ chain was detected indicating that these tumors indeed were not in general of T-cell type. In a single tumor concomitant IgH and TCRβ chain rearrangements were detected, however, this pattern has also been seen in human B-cell malignancies [45]. Surprisingly, rearrangements in Ig loci could be identified in only five of the 31 analyzed tumors (Figure 2A-C), indicating that the tumors are sub-clonal with respect to tumor cell of origin at the molecular level and/or contain significant numbers of non-malignant cells.

Selected tumors with integration in *Cd74 *have been examined by histopathology in connection to our earlier work. As listed in table 2, 26 of the 42 tumors with proviral insertion into the *Cd74 *locus were histopathologically classified and in all 26 cases the hematopoietic neoplasm was characterized to be of B-cell origin.

**Table 2:** Histopathological classification of tumors

Tumor ID and tissue	Histopathology	Tumor ID and tissue- *continued*	Histopathology *-continued*	Tumor ID and tissue- *continued*	Histopathology *-continued*
01-367s	DLBCL*	01-931s	PCP‡	99-34m	ND
00-140n	DLBCL*	01-1069s	PCP‡	99-162c	ND
00-111n	DLBCL*	01-844s	PCP‡	98-1130s	ND
00-216m	DLBCL*	01-898s	PCP‡	99-1159s	ND
01-340s	FBL*	03-462s	PCP‡	01-656s	ND
99-18m	FBL†	01-930s	PCP‡	99-306t	ND
99-191t	FBL†	01-696s	PCP§	01-552s	ND
01-617s	PCP*	01-785c	PCP§	01-720s	ND
99-1276s	PCP*	01-843s	PCP§	01-773s	ND
01-454s	PCP†	01-1067s	PCP§	01-1062m	ND
01-1064s	PCP‡	01-815c	PCP + SMZL§	03-741s	ND
01-671s	PCP‡	99-115c	SMZL†	03-793s	ND
01-700s	PCP‡	99-919m	ND	02-1156m	ND
01-762s	PCP‡	99-1206c	ND	03-1160s	ND

The histopathology of the tumors was evaluated in course of the following studies; *[67], †(Unpublished), ‡[46], and §[68]. DLBCL denotes diffuse large B-cell lymphoma; FBL, follicular B-cell lymphoma; PCP, plasma cell proliferations (consistent with plasmacytomas); SMZL, splenic marginal zone lymphoma; and ND, not determined. Denotation of tissue types as described in Table 1.

### Non-uniformity in Cd74 expression among tumors

The Cd74 mRNA levels in tumor tissue harboring integration in the *Cd74 *locus were assessed by Northern blot hybridizations in order to analyze possible effects of the integrated proviruses on Cd74 expression pattern in the MLV-induced B-lymphomas. As Cd74 is a key component in the process of B-cell development, mRNA from tumors from the same experimental series - but without integration in the *Cd74 *locus - were included as controls, in order to eliminate changes in Cd74 expression levels due to common aspects of MLV infection and/or lymphomagenesis. Sixteen tumors with integration in *Cd74 *were subjected to Northern blot analysis, however, no general pattern of over-expression or down-regulation was evident when compared to controls (data not shown). This observation was also reflected by Western blot analyses of 24 of the 42 tumors when compared to Cd74 expression levels in control tumors (data not shown). The lack of a general mode of transcriptional deregulation could mirror the absence of clonal integrations within the *Cd74 *locus in the tumors and/or be a consequence of a high level of non-malignant infiltrating cells. To assess whether clonal integrations could be identified in the analyzed tumors, Southern blotting was performed with an ecotropic envelope-specific probe. This revealed an absence of clonal integrations on the global scale irrespective of target locus (Figure 2D), and was confirmed for insertions in the locus of interest by Southern blotting with *Cd74*-specific probes (data not shown). The apparent sub-clonal nature of Akv-induced B-lymphomas in inbred NMRI mice has previously been demonstrated and seems to be an inherent feature of these tumor models [46], nevertheless, the existence of CIS in these tumors underscores the effects of insertional mutagenesis [47,48].

In summary, the molecular analyses of end-stage tumor tissues with integrations in *Cd74 *suggest a more complex scenario of Cd74 function in B-cell tumors than oncogenic over-expression or lack of tumor suppressor function.

### Identification of a novel transcript initiated in intron 1 in the *Cd74 *locus

**Figure 3:**
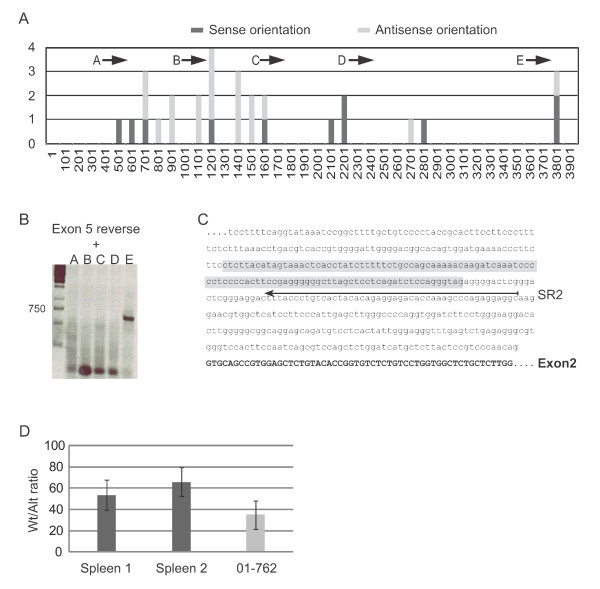
**Identification of a transcriptional initiation region in *Cd74 *intron 1**. **(A) **Overview of the number of integrations within the 3897 bp *Cd74 *intron 1 illustrated in 100 bp windows. The window spanning the first 100 nucleotides in intron 1 is denoted 1 and all other positions are numbered in relation to this. Integrations in co-transcriptional orientation with *Cd74 *are marked by dark grey bars and integrations in antisense orientation are marked by light grey bars. The position of primer A-E applied in the RT-PCR analysis in (B) is indicated. **(B) **RT-PCR analysis on RNA from un-infected NMRI spleen with a *Cd74*-specific exon 5 primer and primer A to E, respectively. **(C) **Results of RLM-RACE analyses, with RNA from un-infected mice, indicating the identified transcriptional initiation region in intron 1 dispersed over app. 100 nucleotides (sequence marked in grey). The position of the primer applied for RT-PCR analyses (SR2) in conjunction with a linker-specific forward primer is indicated. **(D) **Quantitative PCR analyses for detection of the canonical or the novel alternative transcript, respectively, was performed and copy numbers of the two were estimated from copy-number standard-curves derived from amplification on plasmid DNA. The samples included RNA from non-treated NMRI control spleens obtained from two animals (dark grey bars) and RNA purified from tumor 01-762 (light grey bar). The relative ratios of canonical and alternative transcripts in the samples are depicted in the diagram that represents three independent experiments.

Deregulation of cellular genes by proviral insertion can be achieved in different ways. Among other scenarios, proviral elements regulating retroviral transcription are often seen to influence cellular promoters when the provirus is integrated upstream of genes in the antisense orientation. Intron 1 is by far the largest intron in *Cd74 *with its 3897 bp. However, almost all of the antisense integrations in intron 1 (16 out of 18) were found within a window of 950 bp in the 5' end of intron 1 (Figure 3A), suggesting the possible existence of a novel intronic promoter between the proviral integration cluster and exon 2. A forward primer situated in intron 1 just upstream exon 2 and an exon 4 reverse primer was applied in RT-PCRs and in all tumors examined a transcript was identified (data not shown), indicative of the existence of a novel promoter in intron 1. We speculated that if a novel promoter were activated in the tumors, we might also detect this transcript in tissues from un-infected mice and RT-PCR analysis did indeed reveal expression of a transcript containing sequences from the 3' end of intron 1 in spleen from un-infected NMRI mice (Figure 3B). The transcripts initiated in intron 1 consisted of intron 1 sequences extending into exon 2 and with a subsequent canonical splicing pattern downstream of exon 2. The intronically initiated transcript detected in tumor tissues was characterized until exon 5 in which part it was identical in sequence to the novel transcript identified in un-infected tissues.

Furthermore, the amount of intronically initiated transcripts harboring both exon 6 and exon 6b (*i.e*. being p41-like) in tissues from un-infected mice was much lower than the abundance of transcripts harboring only exon 6 (*i.e*. being p31 like) (data not shown), reflecting the relative transcript abundance seen in the human CD74 transcript pool [22].

RT-PCRs with a *Cd74*-specific exon 1 forward primer and several intron 1 reverse primers, of which one was positioned immediately upstream exon 2 did not yield any amplification products indicating that the novel transcript is not an aberrantly spliced isoform of the canonical transcript. In order to identify the transcriptional initiation site of the novel transcript, RNA-ligase-mediated rapid amplification of cDNA ends (RLM-RACE) PCR was performed on RNA purified from two different spleens and PCRs were run with a reverse primer in intron 1 (SR2, Figure 3C). The amplification products were cloned and sequenced and transcriptional initiation sites were in both cases dispersed over a region of app. 100 bp (Figure 3C). In validation of the cDNA synthesis, the canonical transcript was identified by RT-PCR with appropriate primers (data not shown). Thus, in tissues of un-infected mice a transcript is initiated within the 3' part of intron 1 - most likely driven by a broad-peak promoter.

### Expression of the novel Cd74 isoform

Expression of CD74 in the human fetus has been detected in most tissues by immunohistochemistry, where in many cases scattered CD74 staining within non-hematopoietic organs was localized to the interstitium of organ parenchyma [17]. In order to determine the expression pattern of the novel alternative transcript, RT-PCR analysis was performed on different tissues from BALB/c mice. The highest expression of the alternative transcript was seen in spleen, bone marrow, and thymus but the transcript could also be detected in kidney, uterus, ovary, and prostate. Expression of the alternative transcript could however not be detected in cerebrum, cerebellum, testis, heart, liver, skeletal muscle, and lung (data not shown). In contrast to that seen for the alternative transcript, expression of the canonical Cd74 transcript was identified in all tissues analyzed. Accordingly, identification of the canonical transcript in tissues not expressing the alternative transcript indicates differential transcriptional regulation of the two.

To establish the relative abundance of transcripts derived from the canonical upstream promoter and the novel intronic promoter, respectively, a qPCR-based assay was conducted with standards based on absolute amplicon copy-number of the two different amplicons. This revealed the alternative transcript to be a minor isoform with the canonical transcript being 40-60 fold as abundant in un-infected NMRI spleen (Figure 3D). Moreover, in tumor 01-762, which harbors an integration in intron 1 in opposite transcriptional orientation to *Cd74*, the relative wildtype/alternative transcript ratio was slightly shifted towards higher levels of alternative transcripts in accordance with transcriptional activation of the novel isoform by the proviral insertion (Figure 3D, light grey bar).

### A novel Cd74 isoform encoded by the alternative transcript

**Figure 4:**
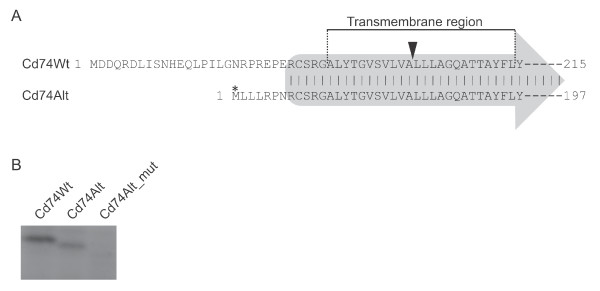
**Identification of a novel Cd74 isoform**. **(A) **Alignment of the N-terminal part of the ORFs in the canonical (Cd74Wt) and the novel alternative isoform (Cd74Alt), respectively. The two ORFs only differ in the N-terminus and are identical from entry into exon 2, which is illustrated in the figure by a grey arrow. The asterisk marks the methionine subject to codon mutation in Cd74Alt_mut in (B) and the arrowhead marks the site of proteolysis. **(B) **Coupled transcription/translation reaction *in vitro *with T7-driven expression of Cd74Wt (lane 1), Cd74Alt (lane 2), and Cd74Alt with the predicted start codon mutated (lane 3, Cd74Alt_mut) in the presence of ^35^S-labelled methionine.

The transcript generated by transcriptional initiation in intron 1 was computationally scanned for open reading frames (ORFs), and interestingly, an ORF was initiated 23 nucleotides upstream exon 2 and terminated in the 5' end of exon 8 encoding a protein of 197 amino acids (aa). Alignment of this novel ORF (of the p31-like exon composition) with that of the canonical p31 Cd74 isoform (215 aa) revealed these to be identical from point of entry into exon 2 sequences - thus, the canonical isoform harbors a 25 aa long unique N-terminal, whereas the first seven aa's from the ORF of the alternative transcript are unique to the novel isoform (Figure 4A). The ORF of the alternative transcripts of the p41-type harboring the additional exon did also align with the canonical p41 ORF in an analogous manner. To verify the usage of the predicted start-codon in production of the novel isoform an *in vitro *coupled transcription/translation assay was performed and confirmed the translation to initiate at the methionine 7 aa's upstream exon 2 (Figure 4B).

### An intronic promoter in intron 1 of the *Cd74 *locus

Expression of an alternative Cd74 isoform driven by a novel intronic promoter in normal tissues prompted the search for *cis*-regulatory elements in the 3' end of intron 1. Initially, the entire *Cd74 *intron 1 sequence was extracted and orthologous sequence chains from rat, human, marmoset, horse, and cow, were obtained and aligned by multiple alignment without gap-penalties with the Chaos and Dialign software [49]. Within this genomic region the largest degree of similarity, excluding the previously described intronic enhancer sequences [19,20], was to be found in the 3' end of intron 1 downstream of both intronic enhancers. This region coincided with the putative location of the novel intronic promoter.

**Figure 5:**
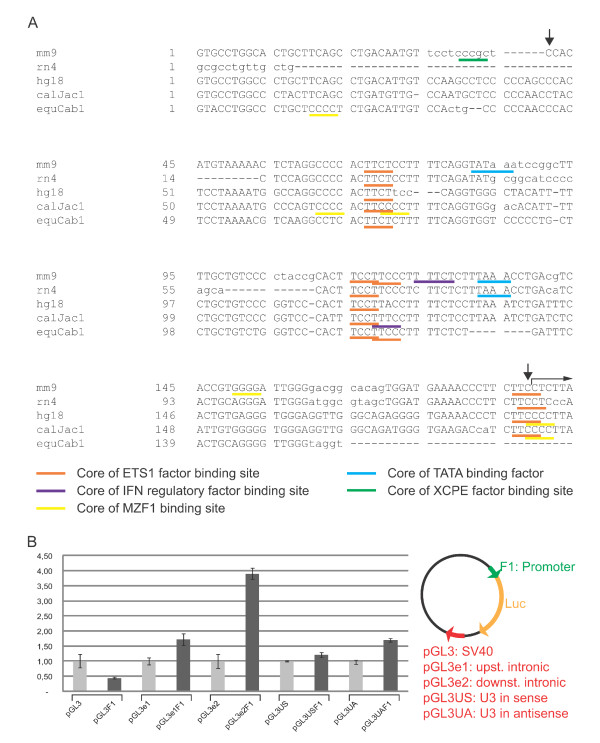
**Identification of a novel intronic *Cd74 *promoter**. **(A) **The sequence upstream of primer SR2 from the RLM-RACE - position of which is indicated in Figure 3C - was applied as query sequence (mm9) to retrieve orthologous sequences from the UCSC genome browser for the indicated species assemblies; rat (rn4), human (hg18), marmoset (calJac1), and horse (equCab1). The retrieved sequences were analyzed by Chaos and Dialign software [49] and the part depicted displayed the highest similarity within the query sequences upstream of the transcriptional initiation region identified by RLM-RACE, the start of which is marked by a horizontal arrow above the sequence. The indicated putative binding site core sequences of transcription factors expressed in the immune system were identified by the MatInspector software [50]. The vertical arrows mark the start and the end, respectively, of the promoter fragment tested functionally in luciferase assays. **(B) **HEK293T cells were transfected with the firefly luciferase reporter pGL3-enhancer (pGL3) or pGL3-derived vectors with the SV40 enhancer in the pGL3-enhancer construct substituted by *Cd74 *intronic enhancers (pGL3e1 and pGL3e2) or by the U3 region of the provirus in sense (pGL3US) or antisense (pGL3UA) orientation respectively, and with or without the promoter fragment (F1) marked by arrows in (A). Luciferase activity of reporters without an inserted promoter are depicted by light grey bars, whereas the activity of equivalent reporters with the F1 promoter region inserted upstream of firefly luciferase are represented by dark grey bars. A plasmid encoding Renilla luciferase under the control of a CMV promoter was co-transfected in all experiments. Luciferase activity was measured 48 hours post transfection, corrected for differences in transfection efficiencies and normalized to the activity of the individual expression vectors with the indicated enhancer but without the F1 promoter fragment which was set to 1.

In order to identify possible conserved transcription factor binding sites within this region in the murine *Cd74 *locus, and in orthologous sequences from rat, human, marmoset, and horse, MatInspector software analysis [50] was conducted with selection parameters ensuring positioning only of predicted binding sites for transcription factors expressed in the immune system with full or nearly perfect match within the core sequence. Several conserved ETS transcription factor binding sites were identified, as well as putative binding sites for myeloid zinc finger 1 (MZF1) (Figure 5A). In the murine sequence two TATA-boxes were identified, however in both cases these regulatory elements were predicted to be situated at a distance to the transcriptional initiation region exceeding that normally observed. In accordance with this, an XCPE1 (X core promoter element 1) motif was identified in the murine sequence, which has been correlated with transcriptional activation of TATA-less promoters in higher eukaryotes [51]. Interestingly, an interferon (IFN) regulatory factor binding site was predicted just downstream a conserved ETS1 site, which in mouse is composed of two consecutive core sequences.

To assess the functional importance of the intronic region harboring the conserved transcription factor binding sites, a plasmid based on the pGL3-enhancer luciferase reporter was cloned in which the region harboring the core sites, the span of which is marked by vertical arrows in Figure 5A, was inserted to drive expression of luciferase as shown in Figure 5B. In the cellular setting of HEK293T cells insertion of the promoter fragment (pGL3F1) resulted in a slight decrease in luciferase activity as compared to the empty vector (pGL3). Previous studies of the *Cd74 *upstream intronic enhancer revealed lack of activity in SV40 promoter-driven transcription indicating a unique match between the cognate promoter and the *Cd74 *intronic enhancer [18,20]. The pGL3 reporter contains an SV40-enhancer and in order to evaluate whether the activity of the novel intronic promoter is enhanced by one of the *Cd74 *intronic enhancers, thereby indicating interdependence between the novel intronic promoter and *Cd74 *cognate enhancers, these were exchanged for the SV40 enhancer (pGL3e1 harboring the upstream intronic enhancer and pGL3e2 harboring the downstream enhancer, respectively). Transcriptional activation experiments clearly showed an increased efficiency of the novel intronic promoter in driving expression of the reporter gene when put in the context of the downstream intronic enhancer, as seen by comparing pGL3e2 (without promoter) and pGL2e2F1 (with promoter) in Figure 5B, while the upstream intronic enhancer only imposed a vague augmentation of promoter activity (compare pGL3e1 with pGL3e1F1 in Figure 5B). Finally, to mimic the situation in tumors with proviral integration in intron 1 reporter constructs were cloned where the SV40 enhancer in pGL3 was exchanged for the U3 transcriptional enhancer region of the provirus in sense (pGL3US) and antisense (pGL3UA) orientation, respectively, in relation to the reporter gene. In this cellular setting, only the enhancer in antisense orientation possessed the capability of increasing the efficiency of reporter gene expression slightly as seen for pGL3UAF1 in Figure 5B.

### IFNγ-responsiveness

**Figure 6:**
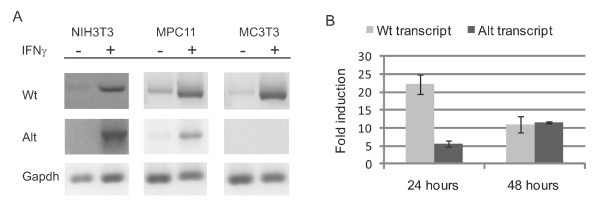
**IFNγ-responsiveness of the novel intronic *Cd74 *promoter**. **(A) **RT-PCR analysis on RNA from NIH 3T3, MPC11, and MC3T3 cells, detecting the canonical transcript initiated in exon 1 (Wt) or the alternative transcript initiated just upstream exon 2 (Alt), respectively, without (-) or with (+) IFNγ-stimulation. The RT-PCRs were performed with 1:10 diluted cDNA as template for detection of the canonical Cd74 transcript and with undiluted cDNA as template for detection of the transcript encoding the novel isoform. Gapdh-specific RT-PCR is included as control. **(B) **Quantitative PCR analyses on NIH 3T3 cells treated with 25 ng/ml IFNγ for 24 and 48 hours, respectively, prior to harvest of RNA. The signal of the Cd74 transcript was normalized to the expression level of TATA-box binding protein in individual cDNA preparations and fold induction compared to un-treated NIH 3T3 cells was calculated on the basis of three independent experiments.

Inspired by the computational analyses indicating the presence of an IFN regulatory factor binding site, we evaluated the IFNγ-responsiveness of the novel promoter in the murine fibroblastic cell line NIH 3T3, the murine myeloma cells line MPC11, and the murine osteoblastic cell line MC3T3, respectively (Figure 6A). The canonical Cd74 transcript was induced in all cell lines tested in accordance with previous studies [52,53], and additionally in NIH 3T3 and MPC11 cells IFNγ-treatment of the cells for 24 hours resulted in induction of the novel alternative transcript. However, in several independent experiments we failed to detect IFNγ-mediated induction of expression of the novel transcript in MC3T3 cells, suggesting differences in promoter activating requirements between the two *Cd74 *promoters. The dynamics of IFNγ-induction of Cd74 transcripts was examined by qPCR detecting the relative transcript abundance in NIH 3T3 cells after IFNγ-treatment as shown in Figure 6B. The canonical Cd74 transcript was induced app. 20 fold after 24 hours incubation with 25 ng/ml IFNγ whereas the amount of intronically initiated transcripts only reached to a level five times higher than that seen in untreated control cells. However, when the cells were treated with IFNγ for 48 hours the levels of fold induction of the two transcripts were comparable (Figure 6B). The relative fold induction in transcript expression was confirmed in NIH 3T3 cells treated with IFNγ at 50 ng/ml for 24 and 48 hours, respectively (data not shown). This data collectively underscore the distinctiveness of the regulatory mechanisms controlling expression of the IFNγ-inducible canonical and alternative Cd74 transcripts, respectively.

## Discussion

### A novel IFNγ-inducible Cd74 isoform expressed from an intronic promoter

The identification of a novel intronic promoter in the *Cd74 *locus was guided by the proviral insertional pattern in this locus. However, the promoter was found to be active in normal un-infected tissues of mice underscoring its activity in the absence of externally introduced transcriptional regulatory elements such as U3 proviral enhancer regions, and pointing towards physiological relevance. The transcriptional control elements for the canonical Cd74 isoform are constituted of a TATA-box containing promoter with common core promoter motifs such as CCAAT-box and Sp1 sites, an upstream enhancer, and two intronic enhancers [18-20]. Notably, the novel promoter, which is situated downstream of both intronic enhancers, does not encompass such common regulatory elements, and transcriptional initiation is dispersed over a region of app. 100 bp, which both are characteristics of TATA-less broad-peak promoters [54]. In this case, the novel promoter is predicted to be under the control of transcription factors (ETS and MZF1) previously shown to be important during hematopoietic lineage specification and controlling proliferative capacity of hematopoietic cells [55,56], and it will be of interest to verify their involvement in transcriptional regulation of the novel Cd74 isoform in functional assays.

The activity of the novel intronic promoter is highly potentiated by the downstream intronic enhancer (Figure 5B). This regulatory unit has been shown to interact with some of the same factors *in vivo *as the upstream intronic enhancer [19], which also to a modest degree influence the activity of the intronic promoter. The U3 enhancer region from the provirus augmented the reporter gene activity only modestly, which was unexpected considering the fact that U3 enhancer insertion is likely to be the activating mechanism applied in the tumors with insertions in antisense orientation. However, the activating potential was evaluated in a cellular setting (HEK293T cells) other than the one in which it is naturally occurring, which is believed to be murine B-cells, and in an artificial system (pGL3-reporter context). We note that only the reporter with the U3 enhancer in antisense orientation compared to the reporter gene was active. This is in accordance with the fact that proviral insertional enhancer activation in tumors irrespective of target locus is primarily seen for insertions in inverse transcriptional orientation to their target gene. In support of a functional connection between the intronic promoter in intron 1 and the *Cd74 *intronic enhancers, none of the insertions in intron 1 in tumors with proviral integrations in *Cd74 *disrupt any of these regulatory elements. This collectively suggests a selection for maintaining functional intronic enhancers with the capability of acting on both the upstream and the intronic promoters. In line with this is that the activity of the 5' intronic enhancer relies on promoter elements other than Sp1-sites and TATA-boxes to promote transcriptional activation of the canonical *Cd74 *promoter [18], which intriguingly characterize the novel promoter.

One of the main transcriptional inducers of Cd74 is IFNγ and both the upstream and the 5' intronic enhancers have been shown to be important for IFNγ responsiveness [20,52,53], whereas the cell-type restricted expression of Cd74 is imposed by the promoter [18]. The MHCII transactivator CIITA is believed to be required for IFNγ induced transcriptional activation of the canonical transcript and both intronic enhancers have been shown to interact with the MHCII transactivator CIITA *in vivo *[19]. Interestingly, ETS1 and MZF1 binding sites are found within the CIITA promoter [57], core sequences of which are predicted to be conserved in the novel intronic promoter. This enables a transcriptionally co-regulated network to act in IFNγ-induction of the canonical as well as the novel Cd74 isoform through the common enhancers and cognate promoters. The novel Cd74 isoform is IFNγ-inducible in agreement with the presence of an IFN regulatory factor binding site in the murine promoter sequence, although the functional impact of this promoter element is not experimentally verified. In addition to a putative contribution to the IFNγ-responsiveness from the promoter itself the downstream enhancer is envisioned to have great influence on the inducibility by IFNγ due to its association with CIITA and its resemblance to the other *Cd74 *enhancer elements. The distinctness of the upstream and the intronic promoters and their different regulation is however manifested through differences in tissues in which the two transcripts are expressed, levels at which they are expressed in the respective tissues, differences in which cell lines the transcripts can be induced upon IFNγ-treatment and the dynamics of transcript prevalence after IFNγ-stimulation.

The novel intronic promoter drives expression of an N-terminally truncated alternative Cd74 isoform, the properties of which currently remain speculative. A detailed characterization at the protein level faces the challenge of deducing conclusive arguments in lack of antibodies specific for one of the isoforms and the close resemblance in size of the proteins but specific functional assays applying e.g. FLAG or HIS-tagged proteins are underway to address some of the questions posed. The novel isoform is however not expected to induce NF-κB mediated transcriptional activation, as it lacks the N-terminus necessary for this activity [13], nor can it be directly involved in motility control in a way analogous to the canonical isoform as this mechanism is a result of interplay between the N-terminal cytoplasmic tail of Cd74 and the actin-based motor protein myosin II [16,58]. However, the formation of the MIF-receptor complex, in which Cd74 constitutes the MIF-binding partner whereas CD44 is the signaling subunit [10,11], is most likely unaffected by the absence of the distant cytoplasmic part, as the transmembrane region is retained in the novel isoform and trimerization is independent of the N-terminus [59]. Additionally, as the two isoforms are expressed concomitantly in different tissues formation of mixed trimers can be envisioned.

### Cd74 is a novel common insertion site in MLV-induced B-lymphomas

Lymphoma induction in mice by MLVs is achieved by injection of infectious particles within the first 48 hours after birth to prevent clearance via an immune response however the state of immunological tolerance seems not to be absolute [60], and it is plausible that the virus may initiate/influence immune signaling prone for Cd74 modulation. Nevertheless, the potential action of immuno-reactive mechanisms during tumorigenesis mediated by the viral infection itself does not weaken the genetic argument brought forward through the identification of *Cd74 *as a CIS for involvement of the *Cd74 *locus in the tumorigenic process.

The Cd74 expression levels, when evaluated on sections of a tumor and not at the single-cell level, could not in general be correlated with the status of proviral insertions within the locus. Interestingly, in tumor 01-762 higher amounts of alternative transcripts relative to the canonical transcript levels were detected by absolute quantification by qPCR in comparison with spleen from un-treated mice. Although tumor 01-762 was the only tumor sample analyzed by this approach and the prevalence of the altered ratio among all the tumor samples therefore remains unknown, it is indicative of transcriptional up-regulation of the novel Cd74 isoform by proviral insertion in this specific tumor. The altered relative ratio of the two transcript forms was however not accompanied by an overall increased Cd74 expression in tumor 01-762, compared to tumors with integrations elsewhere in the genome, as evaluated by Western blotting (data not shown). The general lack of correlation between proviral status and expression levels in end-stage tumors could reflect tumor heterogeneity, which is supported by Southern blotting analyses, and/or that consequences of Cd74 deregulation are manifested at a stage earlier on in the progression of the tumors. Neoplastic transformation as a consequence of a transient signal initiating a positive feed-back loop has recently been reported [61] and this mode of action can also be envisioned in the present model. This does however not exclude possible effects manifested at a pre-leukemic stage in which case it is a formal possibility that cells harboring *Cd74 *proviral insertions are not selected for during tumor outgrowth in analogy to that seen for c-*myb *activation in T-cell lymphomas induced by Moloney MLV in BALB/c mice [62]. Conclusively, although the tumors are sub-clonal with respect to *Cd74 *integrations, the high frequency with which this locus is targeted selectively in B-lymphomas and the obvious non-random distribution of insertion sites within the locus, with respect to position as well as orientation, clearly brings Cd74 forward to be a direct target for deregulation in the B-lymphomagenic process.

### Cd74 isoforms; allies in deed

Deregulation of Cd74 expression is associated with disturbed tumor immune surveillance, which in part is believed to be a result of impaired endogenous tumor antigen presentation [63,64]. Apart from any disturbance imposed by the alternative isoform itself, any changes in canonical Cd74 activity due to its putative association with the novel isoform could be envisioned to disturb the fine-tuned balance of immune surveillance mechanisms holding a tumor at bay. Although no pattern of alternative transcript expression was evident among different tumors with insertions in the *Cd74 *locus (data not shown), this most likely reflect the heterogenic composition of these tumors as suggested by other molecular analyses. Since sequence identity between intronically initiated transcripts from tumor and un-infected tissues was established only from exon 4 and upstream it remains a formal possibility that they differ in their 3' ends. The novel isoform is envisioned to be found on the cell surface in elevated amounts compared to the canonical isoform as it lacks the endosomal localization signals encoded in exon 1 [65], and its deregulated expression due to proviral insertions in the individual cells could increase the pool of available survival receptors prone for MIF stimulation, thereby directly contributing to the tumorigenic process, putatively at the initial stages of malignant transformation. The frequency, position, and orientation of insertions in intron 1 however, clearly underscore the involvement of the novel Cd74 isoform during B-lymphoma development.

## Conclusions

By screening app. 2400 MLV-induced tumors for proviral integration sites we find the *Cd74 *locus to be a novel common insertion site in murine B-lymphomas. Interpretation of the proviral insertion pattern in light of dissecting genomic structures prompted the identification of a novel Cd74 isoform expressed from an intronic promoter in a manner different from its canonical counterpart. Expression of the novel transcript is IFNγ-inducible and reporter assays suggest the activity of the promoter to be under influence of the downstream intronic enhancer.

The essential functions of Cd74 in antigen presentation and B-cell homeostasis together with its recent application as target in immuno-therapy trials, makes a thorough characterization of the protein isoforms of foremost importance. Furthermore, the status of Cd74 as a vital component in the pathogenic process *per se *necessitates a more profound understanding of the pathogenic contribution of Cd74 in the multi-step process of tumorigenesis - in which view expression of an alternative Cd74 isoform only adds to the complexity previously anticipated.

## Methods

### Origin of lymphomas

In previous studies mice of the inbred NMRI strain were infected with the non-acutely transforming ecotropic murine leukemia viruses Akv and SL3-3 and different mutants hereof, primarily harboring mutations within the transcriptional control elements. The set-up resulted in app. 2400 tumors and proviral insertion sites were determined as previously described [47,66]. Tumor samples selected for this study were available from our earlier and unpublished work [41,44,46,48,67,68].

### Southern blotting analyses

Clonal rearrangements in Ig and TCR loci were detected by hybridization of digested genomic DNA extracted from frozen tissues with appropriate probes. Clonal rearrangements in Ig loci were detected by probes derived from the joining regions of the IgH and Igκ [41], respectively, whereas rearrangements within TCR genes were evaluated by two separate probes recognizing either joining region 1 or 2 (J1 and J2, respectively) of the TCRβ chain [69]. The ecotropic virus-specific probe was an envelope *Sma*I fragment from Akv [41].

### RT-PCR

First-strand cDNA synthesis (Fermentas) was made with 3 μg total RNA and an oligo-dT primer followed by PCR amplification. For detection of the novel transcript the following primers were applied:

A: 5' CACCATACAAGTAAGGGCTTTCACAGAT3', B:

5' GGTAACCAGATATGGATTCTTAGA-GCAAT3', C:

5' GAGGGCTGTGTATTCAACAAATCCAT3', D:

5' CGTTATTTAACAACCGCTCA-TTCCAAGC3', E:

5' TGTCACTACACAGAGGAGACACCAAA3', Exon5reverse:

5' TCTGAAGCATCTTAAGAACTCCATGGATG3'. Detection of canonical transcript was performed with Exon1forward + Exon4reverse, while detection of the alternative transcript was performed with Intron1forward + Exon4reverse unless otherwise stated. Exon1forward: 5' CTGTGGGAAAAACTAGAGGCTAGAGC3', Exon4reverse: 5' ACATGGTCCTGGGTCATGTTGCCGTA3', Intron1forward: 5' TGTCACTACACAGAGGAGACACCAAA 3'. Primers for Gapdh were 5' ACCACAGTCCATGCCATCAC 3' and 5' TCCACCACCCTGTTGCTGTA 3'.

### qPCR

For qPCR cDNA was used as template in a SYBR green qRT-PCR reaction using the Platinum SYBR Green qRT-PCR Supermix UDG (Invitrogen) following the manufacturer's recommendations. Reactions were run in triplicates with cDNA corresponding to 30 ng total RNA. For absolute quantification standard curves were derived from amplification on plasmid DNA (expression vectors for the canonical and novel isoform, respectively). The primers applied were one in exon 4 of *Cd74 *(5' ACATGGTCCTGGGTCATGTTGCCGTA3') in combination with a primer specific for the canonical transcript (5' GGGGCTCGAGATGGATGACCAACGCGACC3'), or with a primer specific for the novel transcript (5' ATGCTCTTACTCCGTCCCAACAG3'), respectively. Copy numbers of the two transcripts in the separate spleen samples and tumor 01-762 were estimated from copy-number standard-curves derived from amplification on plasmid DNA.

Relative quantification was done with the Pfaffl method [70] normalizing to the expression level of TATA-box binding protein (TBP) amplified with the primers 5' AGAGAGCCACGGACAACTG 3' and 5' ACTCTAGCATATTTTCTTGCTGCT 3'. Primers in *Cd74 *were 5' GTGCAGCCGTGGAGCTCTGTACAC3' (specific for canonical transcript), 5' GGGGCTCGAGATGCTCTTACTCCGTCCCAACAG3' (specific for novel transcript) in conjunction with 5' ACGCATCAGCAAGGGAGTAGCCATCC3' (*Cd74 *exon 3 reverse primer).

### Identification of 5' transcript ends

Identification of the 5' transcript ends was done using the GeneRacer™ RLM-RACE kit from Invitrogen following instructions provided by the manufacturer. Briefly, 5 μg total RNA from spleen was applied, onto which a GeneRacer™ RNA oligo was ligated to the 5' end. The ligated mRNA was reverse transcribed with a gene-specific primer in exon 5 (5' TCTGGGAAGGTCCCCTTCAGCTGCGGGTACTCCA3') or it was random-primed (primer supplied in the RML-5' RACE kit), respectively. The RNA for gene-specific cDNA-priming was purified from spleen of an inbred NMRI mouse, whereas RNA for the randomly primed cDNA-synthesis was purified from the spleen of a mouse in a backcross breeding program from B6D2F2 to inbred NMRI mice. To amplify 5' ends, amplifications were performed with a forward linker primer and a reverse gene-specific intronic primer (SR2; 5' GCCTCCTCTGGGCTTTGGTGTCTCCTCTGTGTAGTGACAGGGTAA3') in order to identify transcripts initiated in intron 1, and the gene-specific exon 5 reverse primer applied in the cDNA synthesis in order to detect the canonical transcript initiated in exon 1, followed by semi-nested PCR with a nested linker primer. Two μl bulk PCR product was subsequently cloned into the TOPO TA cloning^® ^system (Invitrogen) and sequenced with the provided vector primers M13F and M13R, respectively.

### In vitro transcription/translation

The single-tube *in vitro *transcription/translation assay was performed with the TnT^®^Quick Coupled Transcription/Translation Kit from Promega according to manufacturer's recommendations.

In order to obtain the ORFs in a context with a T7 priming site, the canonical p31 Cd74 ORF and the ORF of the novel isoform (p31-like) were amplified by RT-PCR from oligo-dT-primed cDNA from NMRI inbred spleen RNA and cloned into the TOPO TA cloning^® ^system (Invitrogen). The ORFs were cloned including their 8 upstream nucleotides thereby comprising the naturally occurring Kozak sequence.

### In silico analysis

The sequence spanning intron 1 of *Cd74 *(mm9 assembly of the murine genome at the UCSC genome browser [71]) was extracted. Orthologous sequence chains from rat, human, marmoset, horse, and cow, were obtained and aligned with the murine sequence by global multiple alignment with the Chaos and Dialign software [49,72]. The largest degree of similarity within this region, except regions harboring the intronic enhancers, was from 542 nucleotides upstream exon 2 of murine *Cd74 *and extending until position 202 upstream of exon 2. Thus, a sequence of 317 nucleotides, spanning from 542 nucleotides upstream of exon 2 and until the start of annealing of primer SR2 from the RLM-RACE analysis (which anneals to position 225 → 181 upstream exon 2), was extracted for further analysis. Orthologous sequences from rat (rn4 assembly), human (hg18 assembly), marmoset (calJac1 assembly), and horse (equCap1 assembly), were aligned with the murine sequence by Chaos and Dialign software [49,72]. Transcription factor binding site sequences were identified by usage of the free academic MatInspector license from Genomatix [50,73]. Only transcription factor binding motifs with core similarities higher than 0,85 were considered and furthermore, only transcription factor families expressed in cells of the immune system were included.

### Tissue culture

NIH 3T3 cells were grown in DMEM supplemented with 10% NCS, MC3T3 cells were grown in Mem-a supplemented with 10% FCS, and the MPC11 suspension cell line was cultured in RPMI-1640 supplemented with 10% FCS. In experiments with IFNγ-treatment, cells were grown for 24 or 48 hours in the presence or absence of 25 ng/ml IFNγ, respectively, prior to RNA harvest.

### Luciferase assay

The Dual-Luciferase Reporter Assay System (Promega) was performed according to manufacturer's recommendations. The constructs applied were based on pGL3enhancer (Promega) with or without the promoter inserted in *Xho*I/*Hind*III. The F1 promoter fragment was amplified from BALB/c genomic DNA with the primers 5' TTATATCTCGAGCCACATGTAAAAACTCTAGGCCCCAC 3' and 5' TTATATAAGCTTGAAGAAGGGTTTTCATCCACTGTGC 3'. The SV40 enhancer was exchanged for other enhancers in *Hpa*I/*BamH*I followed by reintroduction of the luciferase polyA site in the *Hpa*I site. Enhancers were amplified with 5' TATAAGTTAACGCTTCCAAGACTGACCAGGCCTTA 3' and 5' TATAATGGATCCGTTTTACTTCCTCCTTTGTACTTCCTCC 3' (upstream enhancer), 5' TATAATGTTAACTCTCCAGCCCTTGGCTTAGGAAATAC 3' and 5' TATAATGGATCCGATAAGTTTGCATCCTGCCTACTCCAG 3'(downstream enhancer), 5' TATAATGTTAACAATGAAAGACCCCTTCATAAGGCTT 3' and 5' TATAATGGATCCCGCCGAGTGTGGGGTTCTTACCCTTTTT 3' (U3 in sense), and 5' TATAATGTTAACCGCCGAGTGTGGGGTTCTTACCCTTTTT 3' and 5' TATAATGGATCCAATGAAAGACCCCTTCATAAGGCTT 3' (U3 in antisense). *Cd74 *enhancers were amplified from genomic BALB/c DNA and the U3 enhancer regions from an Akv-encoding plasmid.

## Competing interests

The authors declare that they have no competing interests.

## Authors' contributions

MP carried out all experimental work except large scale screening for proviral insertion sites, designed and analyzed the experiments, and wrote the manuscript. BW and MW designed and performed the large scale screening for proviral insertion sites. MW and FSP conceived of the study, and FSP contributed to the editing of the manuscript. MP, MW and FSP read and approved the final manuscript.

Bruce Wang died on August 21, 2009.
